# Mutation analysis of *SUOX* in isolated sulfite oxidase deficiency with ectopia lentis as the presenting feature: insights into genotype–phenotype correlation

**DOI:** 10.1186/s13023-022-02544-x

**Published:** 2022-10-27

**Authors:** Jia-Tong Li, Ze-Xu Chen, Xiang-Jun Chen, Yong-Xiang Jiang

**Affiliations:** 1 Department of Neurology, Huashan Hospital and Institute of Neurology, Fudan University, Shanghai, China; 2 National Center for Neurological Disorders, Shanghai, China; 3 Eye Institute and Department of Ophthalmology, Eye and ENT Hospital of Fudan University, 83 Fenyang Rd, 200031 Shanghai, China; 4 Key Laboratory of Myopia, NHC Key Laboratory of Myopia (Fudan University), Chinese Academy of Medical Sciences, 200031 Shanghai, China; 5 Shanghai Key Laboratory of Visual Impairment and Restoration, 200031 Shanghai, China; 6 Human Phenome Institute, Fudan University, Shanghai, China

**Keywords:** Isolated sulfite oxidase deficiency, *SUOX*, ectopia lentis, Neurometabolic disorder, Genotype–phenotype correlation

## Abstract

**Background:**

Isolated sulfite oxidase deficiency (ISOD) caused by sulfite oxidase gene (*SUOX*) mutations is a rare neurometabolic disease associated with ectopia lentis (EL). However, few genotype–phenotype correlations have been established yet.

**Methods:**

Potentially pathogenic *SUOX* mutations were screened from a Chinese cohort of congenital EL using panel-based next-generation sequencing and analyzed with multiple bioinformatics tools. The genotype–phenotype correlations were evaluated via a systematic review of *SUOX* mutations within our data and from the literature.

**Results:**

A novel paternal missense mutation, c.205G > C (p.A69P), and a recurrent maternal nonsense mutation, c.1200 C > G (p.Y400*), of *SUOX* were identified in a 4-year-old boy from 312 probands. The biochemical assays manifested elevated urine sulfite and S-sulfocysteine accompanied by decreased homocysteine in the blood. The patient had bilateral EL and normal fundus, yet minimal neurological involvement and normal brain structure. Molecular modeling simulation revealed the p.A69P mutant had an unstable structure but an unchanged affinity for sulfite, while the truncated p.Y400* mutant showed decreased binding capacity. Genotype–phenotype analysis demonstrated patients with biallelic missense mutations had milder symptoms (*P* = 0.023), later age of onset (*P* < 0.001), and a higher incidence of regression (*P* = 0.017) than other genotypes. No correlations were found regarding EL and other neurological symptoms.

**Conclusion:**

The data from this study not only enrich the known mutation spectrum of *SUOX* but also suggest that missense mutations are associated with mild and atypical symptoms.

**Supplementary Information:**

The online version contains supplementary material available at 10.1186/s13023-022-02544-x.

## Background

Congenital ectopia lentis (EL) refers to the displacement of the crystalline lens from its optical center due to developmental laxity, stretching, and breakage of the zonules[1]. Prognostically, the patient’s vision is threatened by various degrees of refraction error, form deprivation, and detrimental complications such as pupil-block glaucoma and retinal detachment. Congenital EL is mainly inherited, and pathogenic variants can be identified in more than 90% of patients[2]. However, differential diagnosis in the context of EL may not be straightforward. The systematic diagnosis can only be achieved in about 20% patients without further genetic tests[2].

Several inherited metabolic disorders have been associated with EL, including homocystinuria (OMIM: 236,200)[3], hyperlysinemia (OMIM: 238,700)[4], molybdenum cofactor deficiency (MoCoD) (OMIM: 252,150)[5], and isolated sulfite oxidase deficiency (ISOD) (OMIM: 272,300)[6]. Unlike the connective tissue disorders associated with EL, such as Marfan syndrome (OMIM: 154,700)[7] and Weill–Marchesani syndrome (OMIM: 277,600)[8], in which genes coding the structural components of zonules are mutated, the associated metabolic disorders seem to cause EL indirectly through zonule-toxic metabolites in the aqueous humor. However, for a long time, the study of EL in individuals with metabolic disorders has been given insufficient attention.

ISOD is an autosomal recessive inherited metabolic disorder presenting with bilateral EL, significant developmental delay, refractory seizures, microcephaly, and progressive encephalopathy. The causal gene is sulfite oxidase (*SUOX*) (OMIM 606,887), with an open reading frame of three exons and two introns, which is mapped to chromosome 12q13.2 [9]. SUOX, a molybdo hemoprotein located in the intermembranous space of the mitochondria, catalyzes the oxidation of cytotoxic sulfites (SO_3_^2−^) to innoxious sulfates (SO_4_^2−^) in the terminal reaction of the oxidation of sulfur-containing amino acids, such as cysteine and methionine[10]. The word “isolated” when referring to ISOD means the sulfite oxidase defects are solely caused by *SUOX* mutations and do not involve a deficiency of its cofactor (MoCoD). The prevalence of ISOD is challenging to estimate, as to date, only approximately 50 cases and 30 *SUOX* variants have been reported worldwide. Both severe and mild forms of ISOD have been reported; however, the underlying genotype–phenotype correlations remain largely elusive.

In this study, we identified a patient with biallelic *SUOX* mutations from a Chinese cohort of congenital EL by panel-based next-generation sequencing (NGS), and the pathogenicity was analyzed using a series of *in silico* tools. Ophthalmic and neurological examinations showed that the patient had isolated EL with minimal neurological impairment, and biochemical assays confirmed the occurrence of typical changes in metabolites. A systematic review of the genetic and phenotypic features of our patient and previously reported cases was performed in pursuit of the genotype–phenotype correlations.

## Patients and methods

### Patient eligibility and ethics statement

This study was carried out in a manner compliant with the Declaration of Helsinki and authorized by the Human Research Ethics Committee of the Eye & ENT Hospital of Fudan University (ChiCTR2000039132). The establishment of the cohort with congenital EL has been described previously[7, 11]. Only index patients who fulfilled the following criteria were enrolled: (1) diagnosis of EL under slit-lamp microscope, (2) presence of biallelic *SUOX* mutations, (3) availability of clinical information. Patients were excluded if they(1) had a history of ocular trauma or intraocular surgery; (2) suffered from endophthalmitis, end-stage glaucoma, or atrophic eyeball; or (3) harbored mutations in multiple genes related to ocular diseases. Informed consent in written form was obtained from all participants, or their guardians for those under 18 years old.

### Clinical examinations

The medical histories of the patients were carefully investigated, and all of them underwent thorough ophthalmic examinations, neurological assessment, and biochemical testing. Ocular examinations were performed by an experienced ophthalmologist (Y.J.). Slit-lamp examination was performed on both sides under pupillary dilation, and EL was diagnosed if the equator of the lens was visible. Visual acuity was measured as best corrected distance visual acuity (BCVA) (LogMAR) by an experienced optometrist. Intraocular pressure, ocular biometrics, and corneal maps were taken. The anterior chamber, lens, and fundus were imaged. Details of the examination instruments are provided in **Supplementary Table S1**. A physical examination of the nervous system was conducted by an experienced neurologist (X.C.) to include cranial nerve, muscle strength, muscle tone, involuntary movement, movement coordination, sensation, nerve reflexes, pathological signs, and meningeal irritation signs. Any abnormal findings on physical examination resulted in the exam being repeated twice to ascertain its significance. Three-Tesla (3T) brain magnetic resonance imaging (MRI) was performed using the MAGNETOM Vida scanner (Siemens Healthcare GmbH, Germany), which generates T1-weighted imaging (T1WI), T2-weighted imaging (T2WI), and fluid-attenuated inversion recovery (FLAIR) with a high spatial resolution. Urine sulfite levels were obtained using semi-quantitative test strips (QUANTOFIX Sulfite, Macherey-Nagel, Düren, Germany). S-sulfocysteine, cysteine, xanthine, hypoxanthine, and uric acid levels were measured in fresh urina sanguinis. The homocysteine and cysteine levels of blood samples were also measured. All quantitative metabolite tests were conducted in collaboration with MILS Beijing Medical Laboratory (Beijing, China).

### Genetic screening and mutation analysis

Mutation screening was performed using panel-based NGS. Genomic DNA was extracted from peripheral blood samples from a Chinese cohort of congenital EL, as was established in our previous study [7]. The gene panel was produced in collaboration with Amplicon Gene (Shanghai, China) and encompassed 289 genes associated with inherited anterior eye pathologies (**Supplementary Table S2**). The targeted exons and intron–exon junctions were sequenced on the Illumina Novaseq 6000 platform (Illumina Inc., San Diego, CA, USA). Candidate loci were selected if they (1) had coverage of at least 30×; (2) had a minor allele frequency of less than 0.01 (gnomAD, http://gnomad.broadinstitute.org/); (3) were predicted to affect splicing sites (SpliceAI, https://spliceailookup.broadinstitute.org/); and (4) were potentially deleterious, as indicated by more than two *in silico* algorithms (SIFT, http://sift.jcvi.org/; PolyPhen-2, http://genetics.bwh.harvard.edu/pph2/; CADD, https://cadd.gs.washington.edu/; MutationTaster, http://www.mutationtaster.org/). Sanger sequencing was performed to confirm the presence of mutations and co-segregation within family members. The pathogenicity of gene variations was classified according to the classification standards of the American College of Medical Genomics (ACMG)[12]. Protein sequences across species were aligned using the ClustalW algorithm via MEGA7 software. The expression pattern of *SUOX* was obtained from The Ocular Tissue Database (https://genome.uiowa.edu/otdb/) and GTEx (https://gtexportal.org/home/). Inter-database variations were normalized by employing the housekeeping gene *ACTB* as an internal control.

### Structure modeling, dynamic simulation, and molecular docking

Due to the lack of knowledge of the full-length crystal structure of SUOX, the protein structures of SUOX and its mutants were built using AlphaFold (https://alphafold.ebi.ac.uk/). All-atom molecular dynamics simulations were carried out using the LEaP module of the AMBER18 package. The simulated proteins were immersed into a periodic octahedron of pre-equilibrated TIP3P water with at least 10 Å distance around the complexes, and the electroneutrality of the simulation system was balanced with Na^+^/Cl^−^ as appropriate. The energy was minimized through 2500 steps of steepest descent, 2500 steps of conjugate gradient, and 5000 steps without any restraints. The system was gradually heated from 0 to 300 K over 100 ps with position restraints, equilibrated over 100 ps at constant pressure and constant temperature, and re-equilibrated for 100 ps with a weak restraint on the protein backbone. The trajectories were produced for each system in a 30-ns molecular dynamic simulation. The stable trajectory obtained from the simulation was used for molecular docking with AutoDock Vina 1.1.2 software. Water molecules and other undesirable structures were eliminated using PyMOL 2.4 software. The docking box was set to 22.5 × 22.5 × 22.5 Å^3^, the center of which was located using PyMOL2.4 plug-in software. Finally, the best scoring conformation was further visualized.

### Genotype–phenotype analysis

The *SUOX* gene variants in our patients and those in previous reports were all analyzed. PubMed and Web of Science were searched by applying the search terms “(mutation OR variant) AND (SUOX AND (‘sulfite oxidase deficiency’ OR ‘isolated sulfite oxidase deficiency’ OR ‘ISOD’)” from January 1990 to June 2021. Publications were carefully screened according to the inclusion and exclusion criteria. Studies were included if (1) *SUOX* mutation sites were reported and (2) the diagnosis of ISOD was supported by clinical presentations or biochemical findings. Exclusion criteria were (1) duplicated reports, (2) studies lacking clinical manifestations, and (3) reviews summarizing previous mutations. A search for *SUOX* mutations was also conducted using online databases (OMIM, https://omim.org; ClinVar, https://www.ncbi.nlm.nih.gov/clinvar/; and HGMD, http://www.hgmd.cf.ac.uk/ac/index.php/), from which patients were included if they had *SUOX* mutations and were diagnosed with ISOD. Duplicated cases were not included. All variants were aligned with the reference transcript ENST00000394115.6, and those that failed to match any of the *SUOX* transcripts were excluded. Under this search strategy, 19 publications were included in further genotype–phenotype analyses. All processes were performed independently by two authors (J.L. and Z.C.), and any discrepancy in the assessment was resolved by consensus. Mutations were classified as nonsense and frameshift (NF) mutations or missense (M) mutations, and biallelic mutations were subsequently grouped into M + M, M + NF, or NF + NF. The severity of the disease, age of onset, and incidence of comorbidities were compared among the different groups.

### Statistical analysis

The Shapiro–Wilk test was used to test the normality of continuous variables. Continuous variables are presented as means ± standard deviation (SD) for normal distribution and median (interquartile range) for skewed distribution. Numbers (%) are presented for categorical variables. One-way analysis of variance or the Kruskal–Wallis test was used for comparing continuous variables among the three genotype groups, as appropriate. Fisher’s exact test was applied for categorical variables. Differences among the Kaplan–Meier-estimated probabilities of disease onset according to age were compared with Log-rank tests. A two-sided *P* < 0.05 was considered statistically significant. All statistical analyses were computed in SPSS version 25.0 (IBM Corp., NY, USA).

## Results

### Genetic analysis

**Fig. 1:**
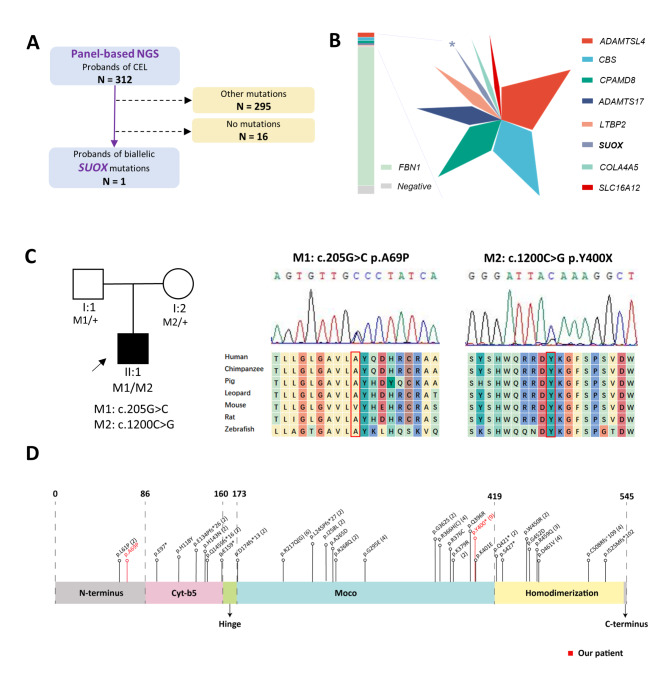
**Biallelic** ***SUOX*** **mutations identified in this study** (A) Brief overview of the enrollment process. One patient with congenital EL and biallelic *SUOX* mutations was identified (B) Proportion of probands harboring biallelic *SUOX* mutations (marked by asterisks). Biallelic *SUOX* contributed to 0.76% of patients in the cohort and accounted for 3.45% of non-*FBN1* mutations (C) Genotype–phenotype co-segregation and protein conservation analysis. The pedigree diagram demonstrates that the identified *SUOX* mutations came from different alleles. The affected member is marked in black, and the proband is indicated by arrows. Sanger sequencing confirmed the compound heterozygosity. The affected amino acid, A69, is conserved in humans, chimpanzees, pigs, leopards, rats, and zebrafish, but not mice, while Y400 is shared by all of the above vertebrates (D) SUOX protein diagram indicating the localization and effects of the existing mutations. The mutations identified in this study are marked in red EL, ectopia lentis; NGS, next-generation sequencing

A total of 312 probands of congenital EL received panel-based NGS and medical evaluation in the Eye & ENT Hospital of Fudan University from January 2016 to Dec 2021 (Fig. 1 A). A novel missense variant c.205G > C (p.A69P) and a recurrent nonsense variant c.1200 C > G (p.Y400*) of the *SUOX* gene were identified in one proband (0.76%) (Fig. 1B). The mutations manifested autosomal recessive inheritance and co-segregated in a *trans* pattern (Fig. 1 C). The affected amino acids are conserved across vertebrates (Fig. 1 C). Both variants had rare allele frequency in different populations. The missense mutation c.205G > C (p.A69P) was predicted to be “deleterious” by SIFT and “possibly damaging” by PolyPhen-2. Therefore, according to ACMG guidelines, the *SUOX* mutation c.205G > C (p.A69P) was classified as “Likely pathogenic”, and c.1200 C > G (p.Y400*) was defined as “Pathogenic”. The biallelic mutations identified in our study were mapped to the protein diagram, together with all *SUOX* mutations reported so far (Fig. 1D). In brief, this patient harbored potentially pathogenic *SUOX* mutations and was suspected of having ISOD.

### Clinical features

**Fig. 2:**
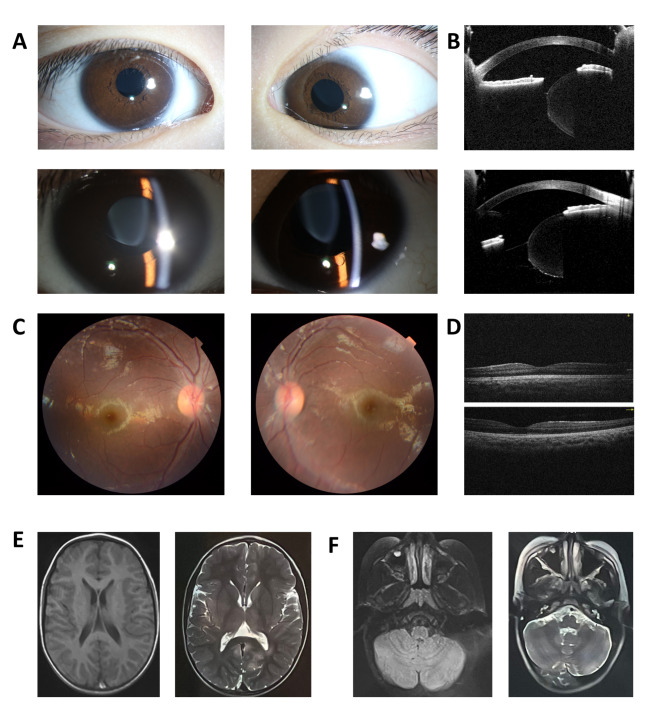
**Ophthalmic and neurological examinations of the patient with biallelic** ***SUOX*** **mutations** (A) Slit-lamp microscope revealed superior dislocation of the lens in the right eye and superior-temporal dislocation in the left eye (B) SS-ASOCT slides manifested dislocated lens and sparse zonules in the right (upper panel) and left eyes (lower panel) (C) Fundus images showed grossly normal macula and optic nerves (D) Fundus OCT images showed no anomalies in macula structure (E) T1-weighted (left) and T2-weighted (right) brain MR images of the basal ganglia plane showed intact brain structures (F) T1-weighted (left) and T2-weighted (right) brain MR images of the cerebellar plane showed no abnormalities OCT, optical coherence tomography; SS-ASOCT, swept-source anterior segment optical coherence tomography; MR, magnetic resonance

The medical history and clinical examination results were carefully reviewed. The patient was a boy aged 4 years and 2 months born to non-consanguineous Chinese parents. He was born with a normal weight (3700 g), and there were no remarkable events during the pregnancy. The family history was also unremarkable. The BCVA was 0.5 LogMAR with − 3.00 DS/−6.00 DC × 150° (OD) and 0.7 LogMAR with − 2.50 DS/−6.75 DC × 145° (OS). EL was diagnosed during regular ophthalmic examinations, and he was then referred to the Eye & ENT Hospital of Fudan University. The lens in the right eye was subluxated upward and that in the left eye was subluxated superior-temporally (Fig. 2 A). Anterior segment optical coherence tomography images showed sparsely scattered zonules at the margin of the dislocated lens in both eyes (Fig. 2B). Fundus photography revealed no evident anomalies (Fig. 2 C), and the macula structure was normal on fundus OCT examination (Fig. 2D). The patient had a short axial length and flattened cornea (**Supplementary Figure S1A**). Topography showed high and irregular corneal astigmatism on both sides (**Supplementary Figures S1B and S1C**). He was suspected of having potential Marfan syndrome until genetic tests revealed the presence of biallelic *SUOX* mutations.

The patient was suspected of having ISOD and was referred to the Department of Neurology of Huashan Hospital, Fudan University, for further assessment. On questioning the guardian into the patient’s medical history, it was declared that the patient had had a fever and had vomited milk 3 days after birth, and these symptoms were relieved after symptomatic treatment. During the first year of his life, he went through several more episodes of fever and vomiting. The post-natal yearly physical examinations revealed no evident delay in his motor or mental development, and no other neurological symptoms were noticed, except for an occasionally unsteady gait. On physical examination, he was of normal height, weight, and head circumference compared to children of the same age. He had no facial dysmorphism but had mild pectus carinatum. His neurological examination revealed no significant abnormalities, except for a slightly unsteady gait and brisk tendon reflex on both upper extremities. His MRI findings were normal at the initial visit (Fig. 2E F). His brain was symmetric, with an intact structure and no atrophy. No obvious increase in signal intensity was found in the bilateral cerebral cortex, globus pallidi, or substantia nigra on T1WI (Fig. 2E, **left panel)** or T2WI (Fig. 2E, **right panel)** images. The bilateral third ventricles and lateral ventricles were symmetrically distributed on both sides of the midline and were of normal size and morphology (Fig. 2E). In addition, although the patient presented with an occasionally unsteady gait, his cerebellum was devoid of structural impairment (Fig. 2 F).

ISOD is generally characterized by neonatal onset of therapy-resistant seizures, severe psychomotor retardation, and early death. In this case, the neurological symptoms were mild and atypical, hence targeted biochemical tests were applied to aid in the diagnosis. The related metabolic pathways are summarized in **Supplementary Figure S2**. A deficiency in sulfite oxidase leads to the accumulation of sulfites and their metabolites, such as taurine, thiosulfate, and S-sulfocysteine, in bypass pathways. An excessive formation of S-sulfocysteine decreases the level of cysteine and homocysteine. The patient showed increased urinary secretion of sulfite and S-sulfocysteine, accompanied with low levels of cysteine and homocysteine in the blood. The normal excretion of xanthine and hypoxanthine in the urine excluded MoCoD, as the lack of molybdenum cofactor would have resulted in a combined deficiency of SUOX and xanthine dehydrogenase (**Supplementary Figure S2**). Therefore, a diagnosis of ISOD was made according to the findings of the above biochemical tests, despite the patient’s minimal neurological anomalies.

### Structural and functional relevance

**Fig. 3:**
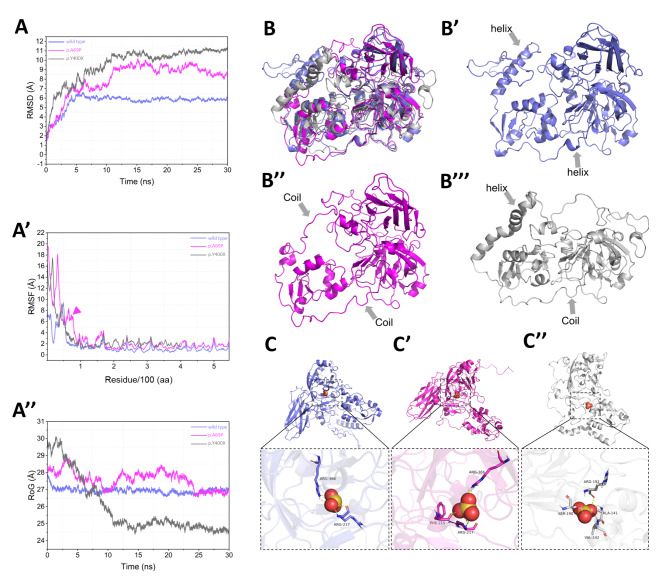
**Structure–function prediction of SUOX protein and its mutants** (A) RMSD plot for the backbone of SUOX wild-type (in violet), p.A69P (in pink), and p.Y400* (in grey) for 30 ns of simulation. Conformation of wild-type protein equilibrated at a level of 6.0 Å after 5 ns and the two mutants equilibrated after 10 ns at higher RMSD. The larger RMSD and later equilibration of the mutants indicate a more unstable structure than that of the wild-type proteins (A’) RMSF graphs for the backbone of SUOX wild-type (in violet), p.A69P (in pink), and p.Y400* (in grey) during the simulation. Both mutants manifested higher levels of flexibility, especially at the N-terminal, than the wild-type. The p.A69P mutant displayed significantly higher RMSF around the mutated residue, indicating more movement than in the wild-type (arrowhead). Plot of p.Y400* terminated at residue 400, consistent with its truncation effects (A’’) RoG plot for the backbone of SUOX wild-type (in violet), p.A69P (in pink), and p.Y400* (in grey) over 30 ns of simulation. The higher RoG indicated more loose conformation in p.A69P, while the p.Y400* is more compact, probably as a result of the reduced size (B-B’’’) Conformation alignment (B) of the wild-type (B’, in violet), p.A69P (B’’, in pink), and p.Y400* (B’’’, in grey) after molecular dynamics simulation. More coil and fewer helix structures (arrows) were found in the two mutants (C-C’’) Molecular docking of the wild-type (C, in violet), p.A69P (C’, in pink), and p.Y400* (C’’, in grey) with sulfite (SO_3_^2−^ ) after molecular dynamics simulation. Wild-type SUOX binds sulfite with Arg366 and Arg217 through a hydrogen bond and salt bridge. p.A69P interacts with additional Phe215. The interaction sites changed to Arg 192, Ser190, Val142, and Phe215 in p.Y400* RMSD, root mean square deviation; RMSF, residue-based root mean square deviation; RoG, radius of gyration

A mild phenotype and relatively late-onset age are rarely seen in ISOD. Thus, our supposition was that the mutations identified in this study probably exerted fewer deleterious effects on the structure and function of the SUOX protein. To test this hypothesis, we built an *in silico* model of SUOX proteins and investigated the mutational effects of p.A69P and p.Y400*. The missense mutation c.205G > C resulted in an amino acid substitution from alanine to proline in the N-terminal of the SUOX protein. The c.1200 C > G nonsense mutation resulted in the substitution of tyrosine with a stop codon in the catalytic domain of the SUOX protein. Root mean square deviation (RMSD) simulation showed delayed equilibration at a higher RMSD for the two mutants, indicating more unstable fluctuations in the mutants than in the wild-type protein (Fig. 3A). From the residue-based root mean square deviation (RMSF) simulation shown in Fig. 3A**’**, the p.A69P mutant was observed to display a significantly higher RMSF around the mutated residue, indicating it had higher levels of flexibility than the wild-type. The plot of p.Y400* terminated at residue 400, consistent with its truncation effects. Both N-terminals of the mutants were less stable than that of the wild-type (Fig. 3 A**’**). The radius of gyration (RoG) plot indicated less compact conformation in p.A69P, while that of p.Y400* was tighter, probably as a result of the reduced size (Fig. 3 A**’’**). After the simulation, the optimized structures were aligned and compared (Fig. 3B-B**’’’**). More coil and fewer helix structures (arrows) were found in the p.A69P mutant, which explained the increased flexibility and decreased compaction observed in the molecular dynamics simulations. Sulfite (SO_3_^2−^) was then docked with the established models (Fig. 3 C-C**’’**), and the p.Y400* mutant showed decreased affinity (− 2.7 kcal/mol) compared with the wild-type (− 3.6 kcal/mol). The binding energy was unaltered in the p.A69P mutant (− 3.6 kcal/mol), even though the binding residues were changed (Fig. 3 C**’**). Overall, *in silico* modeling revealed the unstable structure of the p.A69P mutant and the decreased binding capability of the p.Y400* mutant.

### Mutation spectrum and genotype–phenotype correlations

It is likely that the missense mutation p.A69P can explain the atypical phenotypes observed in the patient; thus, we wondered if missense mutations of *SUOX* generally contribute to less severe ISOD. To test this hypothesis, we reviewed the genotypic and phenotypic features of *SUOX* mutation carriers described in the existing literature. A total of 32 different mutations were identified from 35 pedigrees in this study and previous reports (**Supplementary Table S3**), and the phenotypic spectrum and biochemical tests are summarized in **Supplementary Tables S4 and S5**. Homozygotes accounted for 62.86% of mutations. The most common mutation effect was missense (43/70, 61.43%), followed by nonsense (14/70, 20.00%) and frameshift (13/70, 18.57%), and the most frequent *SUOX* mutation was c.1200 C > G (p.Y400*) (9/70, 12.86%). About 60% of the pedigrees had at least one unique mutation that had no analogs in the literature. Thus, studying the genotype–phenotype correlations of *SUOX* mutations should be helpful in prognostication.

**Fig. 4:**
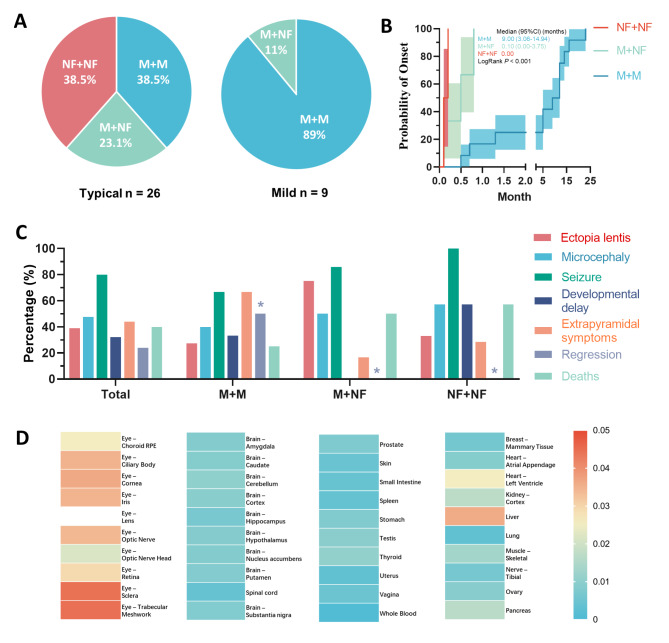
**Genotype-phenotype correlation of ISOD** (A) Proportions of *SUOX* mutations in patients with typical and mild ISOD. (B) Kaplan–Meier-estimated probabilities of *SUOX* mutations on disease onset risk according to age (C) Correlations between *SUOX* mutations and comorbidities, including ectopia lentis, microcephaly, seizure, developmental delay, extrapyramidal symptoms, regression, and death. Asterisks indicate statistically significant changes (D) Heatmap of *SUOX* mRNA expression normalized to *ACTB* in ocular tissues, brain tissues, and other organs. The housekeeping gene *ACTB* was used as the internal reference ISOD, isolated sulfite oxidase deficiency; M, missense mutations; NF, nonsense /frameshift mutations

In the genotype–phenotype analysis, patients were categorized into three groups for further analysis: nonsense/frameshift and nonsense/frameshift (NF + NF) (10, 28.57%), nonsense/frameshift and missense (M + NF) (7, 20.00%), and missense and missense (M + M) (18, 51.43%). The mix of genders (Fisher’s exact test, *P* = 0.338) of the patients was similar among the groups. In total, nearly three-quarters of patients showed a typical clinical ISOD course (26/35, 74.29%), while the others had milder manifestations. Patients with mild ISOD had a higher proportion of M + M mutations (8/9, 88.89%) than M + NF (1/9, 11.11%) or NF + NF mutations (0/9, 0.00%) (Fisher’s exact test, *P* = 0.023) (Fig. 4 A). All patients with NF + NF mutations were typical ISOD cases. The median age at onset was 0.50 months (95%Cl 0.00-1.22). Patients in the M + M group manifested the syndrome at an older age, and the age differences were significant among groups (LogRank test, *P* < 0.001) (Fig. 4B). EL was identified in 7 out of 18 patients (38.89%), the diagnostic age of which varied from 2 months to 8 years old. However, the genotype–phenotype correlation regarding EL was insignificant (Fisher’s exact test, *P* = 0.334). Seizures were the most commonly reported neurological symptoms among ISOD patients (28/35, 80.00%). Patients with M + M mutations seem to be less susceptible to seizures (12/18, 66.67%), though the difference did not reach significance (Fisher’s exact test, *P* = 0.094). Other accompanying symptoms included microcephaly (10/21, 47.62%), developmental delay (8/25, 32.0%), extrapyramidal symptoms (11/25, 44.0%), and regression (6/25, 24.0%). The only significant genotype–phenotype correlation was regression, which was only found in the M + M group (Fisher’s exact test, *P* = 0.017). Ten out of 25 of the patients (40.00%) died from ISOD at ages ranging from 9 days after birth to 9 years old; however, none of the patients with mild or late-onset symptoms died of the disease. Patients with M + M mutations seemed to have a lower rate of mortality (3/12, 25.00%), but it was not significant (Fisher’s exact test, *P* = 0.339) (Fig. 4 C).

### mRNA expression of *SUOX*

Considering the ophthalmic and neurological involvements in ISOD, we wondered if the expression of *SUOX* was more abundant in the ocular and nervous systems. The expression of *SUOX* was analyzed using human RNA expression databases, and *SUOX* exhibited a broad expression spectrum, with expression being most evident in the eyes and liver. In the ocular tissues, *SUOX* expression was prominent in the sclera and trabecular meshwork and lower in the brain and spinal cord (Fig. 4D).

## Discussion

Inherited metabolic disorders associated with EL often come with neurological defects, including mental retardation, epilepsy, and craniofacial deformities. However, these changes alone are not specific enough to facilitate a diagnosis. The discovery of EL can significantly narrow the range of candidate diseases and advocate for targeted biochemical screening and genetic tests. In this study, ISOD was diagnosed in a 4-year-old boy by the joint efforts of ophthalmologists and neurologists. Panel-based NGS revealed the patient had biallelic *SUOX* mutations, the pathogenicity of which were ascertained by biochemical assays. Structural–functional analysis and genotype–phenotype correlation further provided evidence to explain the atypical phenotypes observed in this patient.

A deficiency in sulfite oxidase usually leads to severe and fatal neurological symptoms. The primary pathological signs are cortical swelling, cerebral atrophy, progressive ventriculomegaly, and multicystic encephalomalacia[10, 13]. However, our patient was diagnosed with EL when he was older than 4 years and had normal brain MRI results, which is quite distinct from other reported cases, suggesting the complexity of the mechanisms underlying the neurological impairment. The neuropathology of ISOD has not been fully elucidated as yet. Considering the relatively low expression of SUOX in the nervous system, the severe neurological symptoms were probably due to the vulnerability of the neurons to cytotoxic sulfites. Existing experimental evidence implies there is a disruption to the electronic flow of the respiratory chain due to the accumulation of sulfites and thiosulfites [14, 15]. Sulfites reduce the capacity of Ca^2+^ retention and induce cytochrome c release, interrupting the mitochondrial respiratory process and redox homeostasis, which may eventually cause the dysfunction of brain energy metabolism[15]. This probably explains the presence of neuroradiologic features that are reminiscent of hypoxic ischemic encephalopathy. In addition, it has been hypothesized that the structural similarity of S-sulfocysteine to glutamate and other excitatory amino acids may contribute to the overactivation of N-methyl-D-aspartate receptors, resulting in the refractory seizures of ISOD[10]. Until now, there has been a lack of curative treatments for ISOD, the seizures of which are often pharmaco-resistant and rapidly progressive. Restricting dietary sulfur amino acids has been reported to be useful in milder forms of the disease[16, 17], although spontaneous recovery was also observed in some circumstances[18]. Dietary therapy was not prescribed for this patient, considering his minimal neurological impairment and the relatively high cost of synthetic amino acid mixtures.

A salient feature of ISOD, EL, is identified in about 40% of the patients. However, its prevalence seems to be underestimated because of the likelihood of premature death before the onset of EL and a lack of awareness of ophthalmic examination in some cases. The mechanism underlying EL is mysterious. The speculation is that a defective sulfite oxidase enzyme causes EL indirectly through the action of zonule-toxic metabolites. The zonules are composed of cysteine-rich microfibrils, the intramolecular disulfide bonds of which are susceptible to metabolic changes in the anterior chamber[1]. In cases of homocystinuria, excessive amounts of homocysteine lead to the abnormal formation of disulfide bonds, increasing the susceptibility of zonules to proteolysis[19]. In contrast, in isolated sulfite oxidase deficiency, the disulfide bonds are disrupted, as sulfites are thought to interact with disulfide bonds in vivo to form S-sulfonates, leading to the incompetency of zonules and activation of metalloproteinases[20]. The direction of the EL can be informative for ophthalmologists. The lens tends to dislocate upward in the eyes of patients with Marfan syndrome[21, 22], while interior-nasal and interior-temporal dislocations are commonly seen in homocystinuria and *ADAMTSL4*-related EL, respectively[23, 24]. However, judging from the patient in our study and the limited records in the literature[6], the EL direction of ISOD seems to be indistinguishable from that of Marfan syndrome, which further emphasizes the value of genetic screening, especially for patients with atypical extraocular manifestations.

Panel-based NGS, which was applied to help diagnose our patient, revealed a novel *SUOX* mutation, c.205G > C (p.A69P), and a previously reported nonsense mutation c.1200 C > G (p.Y400*). A literature review showed c.1200 C > G (p.Y400*) to be the most frequent mutation of the *SUOX* gene, the biallelic mutations of which are associated with typical ISOD[9, 25]. This nonsense mutation was localized to the C-terminus of the molybdenum-cofactor-harboring domain and was predicted to result in the premature termination of SUOX and a decrease in its binding affinity to sulfites. Conversely, the missense mutation c.205G > C (p.A69P) was present in the N-terminus domain and induced unstable conformation but had no effect on sulfite affinity. Thus, we speculated that the atypical phenotypes observed in our study can be attributed to c.205G > C (p.A69P). Further genotype–phenotype analysis revealed that ISOD patients with less deleterious mutations, such as missense ones, were more likely to have a mild involvement with late onset. However, the correlations between comorbidities and *SUOX* mutations were vague. The only significant correlation was seen for regression, which was exclusively found in the M + M group. However, the earlier onset age in patients with M + NF and NF + NF mutations is likely to interfere with the evaluation of regression symptoms. Furthermore, the premature death of those with severe forms of ISOD might also hinder the evaluation of EL, developmental delay, and extrapyramidal symptoms, and would probably conceal any potential genotype–phenotype correlations.

The conclusions we drew from this study should be considered within the context of its limitations. First, only one patient with ISOD was identified in our cohort, probably due to the disease’s rarity. Thus, the genotype–phenotype analysis largely relied on systematic review data, the interpretation of which was limited by the potential reporting bias and incomplete records for some cases. Second, the progression of the neurological symptoms and potential changes to brain structure demand further exploration. Third, we tried to correlate the biochemical findings with the genotypes and phenotypes, yet few qualified data were collated, and there were inconsistencies in the assays and settings among the studies. Thus, whether the biochemical results can be used to infer phenotypic severity warrants further investigations. Despite the above weaknesses, to our knowledge, this is the first recorded case of an ISOD patient presenting with EL and normal brain structure at a relatively old age, reaffirming the importance of genetic testing and multidisciplinary collaborations in diagnosing congenital EL.

In conclusion, biallelic *SUOX* mutations are one of the causes of congenital EL. Although ISOD can be recognized by the advent of severe seizures, microcephaly, and developmental delay by neurologists and ophthalmologists, late-onset cases with EL as the indicative symptom may also be observed. The phenotypic diversity of patients with ISOD can be partially explained by their genetic heterogeneity.

## Electronic supplementary material

Below is the link to the electronic supplementary material.

Supplementary Material 1

Supplementary Material 2

Supplementary Material 3

Supplementary Material 4

Supplementary Material 5

Supplementary Material 6

Supplementary Material 7

Supplementary Material 8

## Data Availability

All data relevant to the study are included in the article or uploaded as supplementary information. Both variants and the phenotype of the patient reported in this study have been submitted to Global Variome shared LOVD under the owner name of Xiangjun Chen by default licenses using a Creative Commons Attribution 4.0 International License(https://databases.lovd.nl/shared/individuals/00411160).

## Abbreviations

ACMG: American College of Medical Genomics
BCVA: best corrected distance visual acuity
EL: ectopia lentis
FLAIR: fluid-attenuated inversion recovery
ISOD: isolated sulfite oxidase deficiency
MoCoD: molybdenum cofactor deficiency
MRI: magnetic resonance imaging
NGS: next-generation sequencing
OCT: optical coherence tomography
RMSF: residue-based root mean square deviation
RoG: radius of gyration
SD: standard deviation
SS-ASOCT: swept-source anterior segment optical coherence tomography
SUOX: sulfite oxidase
T1WI: T1-weighted imaging
T2WI: T2-weighted imaging
